# Self-dehumanization and other-dehumanization toward students with special educational needs: examining their prevalence, consequences and identifying solutions—a study protocol

**DOI:** 10.1186/s40359-023-01178-3

**Published:** 2023-04-27

**Authors:** Kuen-Fung Sin, Lan Yang, Frank Tian-Fang Ye

**Affiliations:** 1 Centre for Special Educational Needs and Inclusive Education, The Education University of Hong Kong, Tai Po, Hong Kong SAR People’s Republic of China; 2 Department of Curriculum and Instruction, The Education University of Hong Kong, Tai Po, Hong Kong SAR People’s Republic of China; 3 Department of Applied Social Sciences, The Hong Kong Polytechnic University, Kowloon, Hong Kong SAR People’s Republic of China

**Keywords:** Dehumanization, Special education, Students, Hong Kong

## Abstract

**Background:**

Students with special educational needs (SEN) often face dehumanization, which negatively impacts their mental health, daily functioning, and educational outcomes. This study seeks to address the research gap in dehumanization literature by examining the prevalence, dynamics, and consequences of self-dehumanization and other-dehumanization among SEN students. Moreover, by utilizing psychological experiments, the study aims to identify potential intervention strategies and make recommendations to minimize the negative psychological consequences derived from the dual model of dehumanization.

**Methods:**

This two-phase, mixed-methods study incorporates cross-sectional surveys and quasi-experimental designs. Phase 1 investigates the self-dehumanization of SEN students and other-dehumanization from non-SEN peers, teachers, parents, and the public. Phase 2 involves four experimental studies to evaluate the effectiveness of interventions emphasizing human nature and uniqueness in reducing self-dehumanization and other-dehumanization of SEN students, as well as their associated negative consequences.

**Discussion:**

The study fills a research gap by examining dehumanization in SEN students, applying dyadic modeling, and identifying potential solutions to ameliorate dehumanization and its negative consequences. The findings will contribute to the advancement of the dual model of dehumanization, increase public awareness and support for SEN students in inclusive education, and promote changes in school practice and family support. The 24-month study in Hong Kong schools is expected to provide significant insights into inclusive education in school and community settings.

## Background

In Hong Kong, inclusive education for students with special educational needs (SEN) in mainstream schools has been fast developing since 1997. In 2019, SEN students made up 7.8% (22,980) of the total in primary schools, and 8.6% (22,380) of the total in secondary schools [1]. Compared to the relatively stable number of students in special schools (7,700), the population of SEN students in mainstream schools has increased 34% over the past five years. The Education Bureau (EDB) has been promoting the Whole School Approach to Integrated Education and implemented considerable resources and interventions to promote mutual respect of individual differences, as well as cater for student diversity [2].

However, as minority group members, SEN students are always the targets of discriminatory treatment. Previous studies have found that SEN students have more negative views on their relationship with teachers and peers, compared to non-SEN students. They are also more likely to get laughed at and bullied in schools [3]. Studies in Hong Kong primary schools showed that SEN students were sometimes labeled and ignored by teachers [4]. Such alienation and rejection have been found to start as early as elementary school [5].

The discriminatory experience can be reciprocal and disastrous in school settings. For example, as both SEN students and teachers view their relationship as unsatisfactory, it negatively impacts both sides [6]. Such a relationship is reflected in the emotional experience and social interaction of SEN students, and consequently affects their academic performance [7], and psychological and behavioural functioning [8].

With an increasing number of SEN students enrolled in mainstream schools, the aforementioned problems will be aggravated. Considering this situation, there is an urgent call for understanding the underlying mechanism of the prejudice towards SEN students in inclusive settings. It is worthwhile examining the fundamental humanness attribution error, dehumanization, as the foundation for understanding such prejudice. Furthermore, based on the mechanism of dehumanization, teachers have to develop effective intervention strategies to battle discrimination in classrooms.

### The dual model of dehumanization

The booming psychological research into attribution of humanness offers an integrative approach to examining the discrimination. The denial of humanity can be the root of prejudice toward people with disabilities. Dehumanization, the tendency of attributing fewer human characteristics to others and perceiving others as less human, has been a topic of great interest over the past few years [9]. It is a pervasive prejudicial, and discriminatory cognitive process that exists in people’s daily life [10, 11]. Dehumanization can be expressed in blatant forms (e.g. “they look like animals”), or subtle forms (e.g. “people with ADHD cannot enjoy peace”) and can be easily activated in a variety of contexts in people’s daily lives [12–15]. Viewed as somewhat lacking in human characteristics, people who are dehumanized are open to social exclusion and hostility [16].

Early research conceptualized dehumanization primarily in the context of morality [17, 18]. Recent development in the field has enriched the understanding of dehumanization as a border conceptual framework that encompasses attributing uniquely human emotions [19, 20], warmth and competence [21, 22], mental states [23], and personality traits [24].

According to the Dual Model of Dehumanization [13, 16, 24], people attribute a lack of humanness in others through viewing them as more similar to animals (lacking Human Uniqueness, HU), or viewing them as more similar to robots (lacking Human Nature, HN). This two-dimensional model of humanness has been broadly adopted in recent dehumanization research, and its universalism and implications are supported by a substantial amount of empirical evidence [9, 25]. Thus, the dual model (lacking HU and HN) will be helpful to understand the nature of dehumanization.

### Consequences of dehumanization

Many studies have demonstrated the link between dehumanization and harmful consequences [9, 26]. Some have profound implications in inclusive settings. For example, when criminals are judged by the public, those who are considered to lack humanness are more likely to receive harsher punishment [27]. Other studies have found similar effects for earthquake victims, those who are dehumanized are less likely to receive humanitarian aid [28]. This paradigm could be relevant for teachers and peers to make decisions when a SEN student conflicts with or bullied by non-SEN peers. In a study, teachers’ dehumanization of minority students is further found to be predictive of their discrimination towards and harsher treatment of those students [29]. These consequences highlight the importance of understanding dehumanization in inclusive education, and the need to develop interventions reducing its harmful consequences in SEN students’ daily life.

Apart from being labelled in schools and classrooms, SEN students may be dehumanized in a way similar to people with disabilities. Some studies examined the dehumanization of adults with intellectual and developmental disabilities and found that they are seen as lacking human uniqueness by professionals in day-care centres [30]. Another study revealed that greater dehumanization of people with Autism or Down’s syndrome predicted stronger prejudice towards and reduced social policy support for this group [31]. Moreover, people with mental disorders are dehumanized even more severely than ethnic minorities and immigrants, which further relates to various kinds of stigma (e.g., social distance) within society [32]. These findings highlight the unneglectable existence of the dehumanization phenomenon regarding these vulnerable groups, as well as its harmful consequences.

### Self-dehumanization by SEN students

The aforementioned dehumanization studies have mostly been through the lens of “perpetrator” (other-dehumanization), while the essential and ultimate goal of understanding dehumanization is to help the victims. Therefore, it is worthwhile examining the prevalence and nature of self-dehumanization from the perspective of SEN students. The self-dehumanized persons were at risk of negative mood emotions, pessimistic mental states and aversive self-awareness [33]. Ignoring the self-dehumanization of SEN students may lead to severe consequences, such as learned helplessness and degraded mental health [3]. Experimental studies among adults have found that a prolonged experience of powerlessness results in self-dehumanization [34]; involvement in unethical behaviours leads to self-dehumanization, which in turn leads to continued unethical behaviours [35]. Researchers and educators should pay attention to the causes and intervene appropriately to minimize the adverse consequences.

Dehumanization among children or self-dehumanization among people with disabilities is rarely investigated [11]. It may be probable that many studies adopted the common paradigm that involves measuring abstract personality traits and human characteristics [25], which is not suitable for persons without a sense of agency, the capability of understanding abstract thinking, or complex emotions. Thus, the immediate objective of this research study is to develop and evaluate a new measure suitable for SEN students.

### The family context of dehumanization

**Fig. 1:**
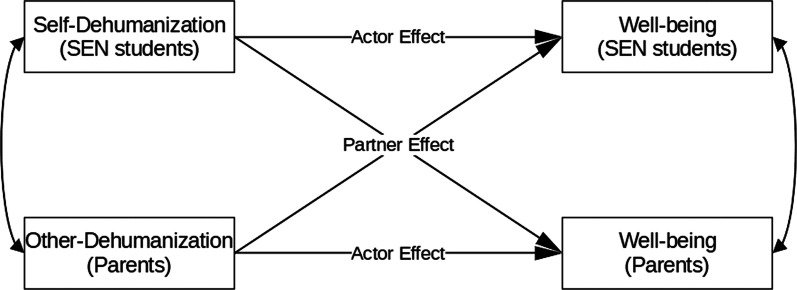
A sample dyadic relationship of dehumanization on well-being between SEN students and their parents, illustrated in the Actor-Partner Interdependence Model

**Fig. 2:**
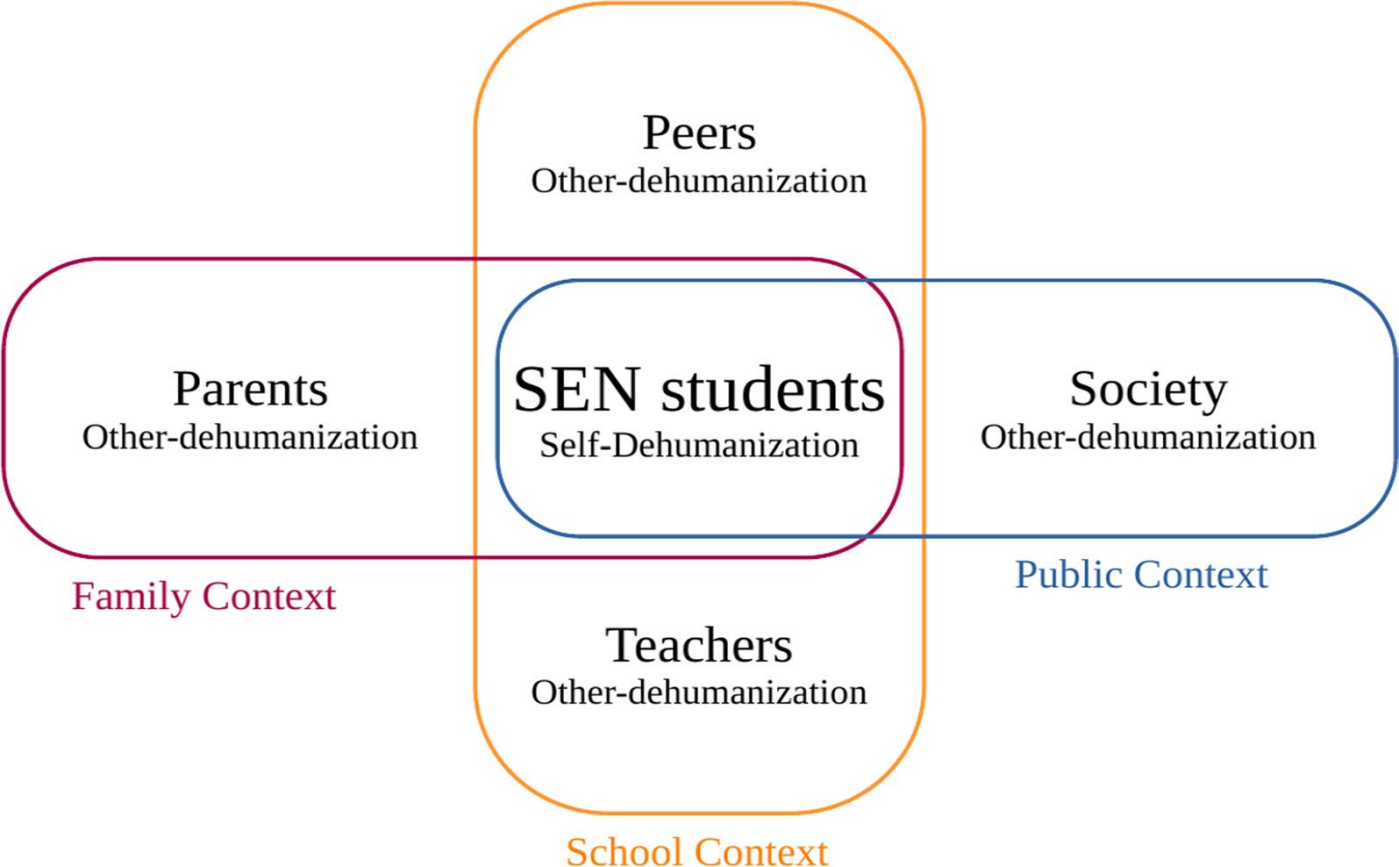
Conceptual framework of the study

As summarized above, both other-dehumanization and self-dehumanization bring challenges in public health and education contexts. Beyond researching each target population separately, it is necessary to consider the interpersonal relationships, and look into the actor-partner effect of dehumanization, for example, the dyadic effect of SEN students and non-SEN peers. This is no doubt a prominent question. After all, establishing connections with society is at the core of inclusive education. In the literature, some studies have found such a dyadic effect of dehumanization in terms of sexual objectification and unequal working environments [36]. Again, inclusive education has received little attention. Thus, examining the dyadic relations will enrich the understanding of interpersonal and contextual aspects of dehumanization (demonstrated in Fig. 1). For SEN students, unfolding dehumanization in the family and school contexts will facilitate more effective intervention strategies and improvements in education policies (demonstrated in Fig. 2).

Different categories of SEN, e.g., ADHD, ASD, SpLD, are identified in schools [3]. Non-SEN individuals may attribute humanness to them based on subtle differences in stereotypes or experience of interpersonal contact with SEN students. For example, students with ADHD may be seen as overly expressive in secondary emotions, thus high in HU. Students with ASD may be seen as lacking HU. Given the limited literature in the field, it is necessary to take the post-hoc approach by considering the SEN type, and analyse the differences within the empirical data. The examination of the dehumanization among SEN students, as a minority group in inclusive setting, is thus the focus.

### Research gaps

Firstly, previous dehumanization studies have mostly focused on ethnic and racial groups, even though topics on gender inequality and social minorities have begun to receive growing attention [9, 37]. However, little attention has been paid to inclusive settings. In particular, no research has been conducted among SEN students. Thus, given the growing number of SEN students in Hong Kong schools, it is important to understand the prevalence and nature of self-dehumanization and other-dehumanization among these students.

Secondly, to date, there has not yet been an available dehumanization measurement tool for SEN students, or SEN individuals in general. Thus, the immediate objective of this study is to develop and evaluate a new measure suitable for the target population.

Thirdly, so far, dehumanization studies simultaneously taking the perpetrators’ and victims’ perspectives (e.g., self-dehumanization vs. other-dehumanization) are rare in the field [36]. There is a research gap in investigation of the potentially reciprocal relationship, for instance, in a family context that involves SEN students and caregivers, or in a classroom that involves SEN students and non-SEN peers. The psychological consequences of these relations will be examined through dyadic modelling.

Lastly, to understand the nature and implications of the dehumanization of SEN students, it is necessary to develop effective strategies to ameliorate dehumanization and its negative impact. Therefore, the study will incorporate previous findings and conduct programs to explore this possibility. Furthermore, it will be appealing to identify practical interventions in a way that is up-to-date, attractive, and trending in popular culture among youngsters, e.g., short video clips that re compatible with Instagram.

### Assumptions and research questions of the study

With the mixed methods approach, the study is with two phases. The *first phase* will gain a deeper understanding of the dehumanization of SEN students through the lens of victims (self-dehumanization of SEN students), close relations (other-dehumanization from non-SEN peers, teachers, and parents), society (other-dehumanization from public), and lead to the *second phase,* in which we will conduct experimental studies for developing effective intervention strategies to reduce dehumanization.

In Phase 1, we will conduct two cross-sectional survey studies to tap into the dehumanization of SEN students: Study 1a will recruit SEN students from mainstream schools in Hong Kong and examine the nature and impact of self-dehumanization. In addition, we will recruit non-SEN peers, teachers and parents of the SEN students mentioned above, and examine the impact of other-dehumanization from close contacts. Study 1b will recruit non-SEN students (enrolled in schools or classes without SEN peers) other than those mentioned in Study 1a, their parents, as well as university students, to investigate the nature and effects of other-dehumanization that SEN students receive from the public.

### Research questions in Phase 1

1. In what ways do SEN students dehumanize themselves? Is self-dehumanization related to their mental health and daily functioning in schools (e.g., learning effectiveness, social interaction)?
2. In what ways do non-SEN peers, teachers, and parents dehumanize SEN students? Is other-dehumanization related to prejudice towards SEN students?
3. Is there any relationship between the self-dehumanization of SEN students and the other-dehumanization from school peers, teachers, and parents? How do such relations interact with well-being and school functioning on both sides?
4. In what ways does the public dehumanize SEN students? How does the other-dehumanization relate to prejudice towards SEN students?

In Phase 2, aiming at ameliorating the self-dehumanization and other-dehumanization, we will conduct four experimental studies to understand the underlying mechanism: Study 2a & 2b will adopt the approach used in previous research [38], directly manipulating perception of humanness, and evaluate its effects on the self-dehumanization and other-dehumanization. Study 2c and 2d will incorporate the findings from studies in Phase 1 and Study 2a & 2b, produce video clips as experimental stimuli, and investigate the priming effects of watching these videos on dehumanization among participants.

### Research questions in Phase 2

1. Will or to what extent might the self-dehumanization of SEN students and its negative impact be reduced by presenting information emphasizing the human nature/human uniqueness (e.g. by reading relevant materials or watching a video clip)?
2. Will or to what extent might non-SEN peers’, teachers’, and parents’ other-dehumanization of SEN students and its negative impact be reduced by presenting information emphasizing human nature/human uniqueness (e.g. by reading relevant materials or watching a video clip)?
3. Will or to what extent might the other-dehumanization of SEN students from the public and its negative impact be reduced by presenting information emphasizing human nature/human uniqueness (e.g. by reading relevant materials or watching a video clip)?

In summary, in Phase 1, we will explore the prevalence and nature of self-dehumanization of SEN students. The findings are predicted to be negatively related to the mental well-being and school functioning of SEN students. We will also investigate other-dehumanization from non-SEN school peers, teachers, and parents, and test whether dyadic relations exist between SEN students and their close relations (e.g., if there’s a dyadic relation of dehumanization on well-being between SEN students and their parents; see Fig. 1). In addition, among the public, it is expected that the other-dehumanization is to be positively related prejudice and reduced policy support.

In Phase 2, by incorporating the findings from Phase 1, we expects to find a negative impact of lacking humanness priming compared to having humanness priming (e.g., after reading the lacking humanness materials, participants demonstrate greater prejudice compared to those who read having humanness materials). In addition, it is expected to identify the positive impact of watching video clips emphasizing SEN students’ humanness.

## Methods

### Participants

Phase 1: for Study 1a, we will recruit SEN students, their parents, teachers, and non-SEN peers (i.e., classmates of SEN students) in mainstream secondary schools in Hong Kong that implement inclusive education. For Study 1b, we will recruit non-SEN students in secondary schools in Hong Kong other than those who participated in Study 1a, their parents, as well as non-SEN university students. We have conducted power analysis to calculate the ideal sample size. We assume a small-to-medium effect size r = 0.21 [39] for associations of dehumanization and other variables, 175 participants are required to obtain 80% power with 5% ɑ error rate for a two-tailed Pearson correlation test. We also conducted Monte Carlo simulation for identifying a 10-item measurement model with moderate factor loadings, lambda = 0.60 [40], 150 participants are required to obtain good model fit indices (CFI > 0.90, RMSEA < 0.06, SRMR < 0.06), which is less than 175. We expect the data attrition and invalid answers to be 10%. Thus, for each target population we aim to recruit 193 (175 × 1.1) participants, except for school teachers (we aim at 50 for practical reasons). Hence, in total we aim to recruit 629 participants (193 SEN students + 193 peers + 193 parents + 50 teachers) in Study 1a, and 386 (193 non-SEN students + 193 parents and university students) participants in Study 1b.

Phase 2: In Study 2a and 2c, we will recruit SEN students, their parents, teachers, and non-SEN peers to participate in the experiments. In Study 2b & 2d, we will recruit non-SEN university students and their parents to participate. However, we may not be able to recruit enough teachers in Study 2a and 2c. Therefore, we will focus on the other three groups. Power analysis suggests that 180 participants are required to obtain 80% power with 5% ɑ to find medium-size differences between 4 conditions (one-way ANOVA, f = 0.25), and 160 participants are required for 3 conditions. Thus, we aim to recruit 540 participants (180 SEN students + 180 peers + 180 parents) in Study 2a, 180 non-SEN participants in Study 2b, 480 participants (160 SEN students + 160 peers + 160 parents) in Study 2c, and 160 non-SEN participants in Study 2d.

### Procedure and instruments

**Fig. 3:**
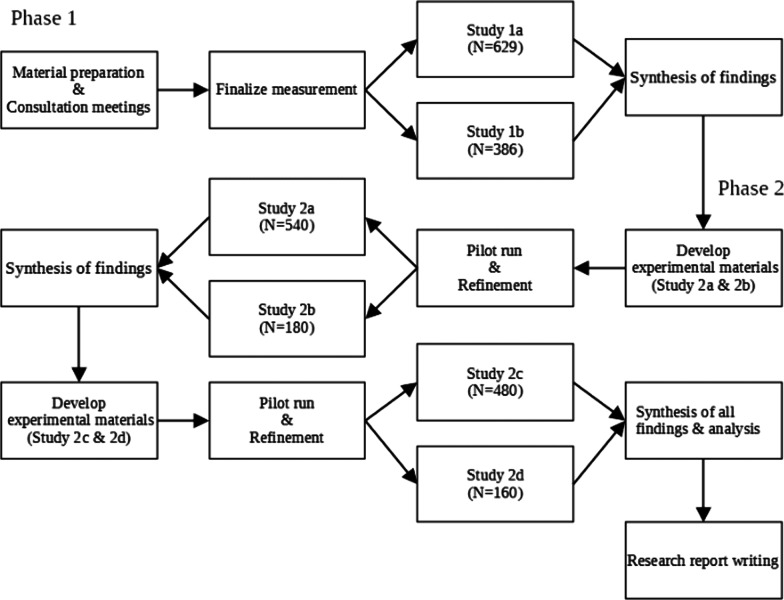
Workflow of the study

For clear presentation, the key measures discussed are summarized in Table 1 and the workflow of the study are presented in Fig. 3.

**Table 1:** Summary of key measures in the study

Participants	Measures used	Analysis focus
*Phase 1: Study 1a*		
SEN students (N = 193)	*Self-dehumanization* *Satisfaction with Life Scale* *School Engagement Scale* *Academic and career self-efficacy*	Within-group and between-group differences of dehumanization; Associations of dehumanization and outcomes; Dyadic effects
Non-SEN peers of SEN students (N = 193) Teachers of SEN students (N = 50) Parents of SEN students (N = 193)	*Other-Dehumanization of SEN students* *Prejudice towards SEN students* *Social Distance Scale* *Subjective Well-being* *School Functioning*^(1)^	
*Phase 1: Study 1b*		
Non-SEN students (in schools or classes without SEN peers) (N = 193) Parents of non-SEN students and University students (N = 193)	*Other-Dehumanization of SEN students* *Prejudice towards SEN students* *Social Distance Scale* *School Functioning*^(1)^ *Public Policy Support*	Within-group and between-group differences of dehumanization; Associations of dehumanization and outcomes
*Phase 2: Study 2a & 2b*		
2a) SEN students (N = 180) Non-SEN peers of SEN students (N = 180) Parents of SEN students (N = 180) 2b) Non-SEN individuals (N = 180)	*Baseline Dehumanization Measure* *Experimental Manipulation (Reading: lacking* *HU, having HU, lacking HN, having HN)* *Manipulation check questions* *Prejudice towards SEN students* *Social Distance Scale* *Public Policy Support* *Subjective Well-being*	Between-group differences of dehumanization and outcomes
*Phase 2: Study 2c & 2d*		
2c) SEN students (N = 160) Non-SEN peers of SEN students (N = 160) Parents of SEN students (N = 160) 2d) Non-SEN individuals (N = 160)	*Baseline Dehumanization Measure* *Experimental Manipulation (Video: HU* *emphasis, HN emphasis, control)* *Manipulation check questions* *Prejudice towards SEN students* *Social Distance Scale* *Public Policy Support* *Subjective Well-being*	Between-group differences of dehumanization and outcomes

^(1)^For teachers, the questions assess their perceived school functioning of SEN students and non-SEN students in their class; for parents, the questions assess their perceived school functioning of their kids

#### Phase 1

In Study 1a, SEN students will be invited to complete the Chinese version of the dehumanization measure with instruments described below. Due to anticipated difficulties in reading and understanding the items, the questionnaire will be designed in short form, with minimal complexity in vocabulary and questionnaire formats. Rating scales will be designed to be SEN-friendly. Likert scale points may be replaced with shapes and emojis. Prior to the survey administration, translation and back-translation will be conducted with the consultancy from SEN experts in CSENIE.

##### Self-dehumanization

A 10-item short-form dehumanization measure assessing self-perceived human uniqueness and human nature will be adapted from previous research, Cronbach’s ɑs > 0.8 [41]. The original measure has been validated across cultures and within various populations [25]. Participants will rate the extent to which they perceive the humanness traits best describe themselves (e.g., warm, shy [reverse-coded]). Items are rated on a 5-point Likert scale (1 = not at all, 5 = very much).

##### Subjective well-being

Students’ subjective well-being will be assessed by the widely used 5-item Satisfaction with Life Scale (SWLS) [42]. Previous studies suggest SWLS is reliable, Cronbach’s ɑs > 0.8 [43]. Sample items include, “In most ways my life is close to my ideal” and “I am satisfied with my life.” All question items are rated on a 7-point Likert scale (1 = strongly disagree, 7 = strongly agree).

##### School functioning

A 19-item School Engagement Scale [44] will be used to assess students’ daily functioning at school. The Chinese version that has been validated in the Chinese context in previous research, Cronbach’s ɑs > 0.8 [45], will be used. Sample items include “I feel happy in school” and “I pay attention in class”. In addition, to assess students’ academic and career self-efficacy, we will adapt a 10-item measurement previously validated among SEN students in Hong Kong [46]. All items are rated on a 5-point Likert scale (1 = not at all, 5 = very much).

##### Demographic information

Information regarding the school profile, students’ grade, gender, age, SEN type and academic level will be collected.

In the meantime, the non-SEN peers, teachers, and parents of SEN students will be invited to complete a Chinese version of the dehumanization measure with instruments described below. Translations and instructions will differ from the version for SEN students, depending on the target population.

##### Other-dehumanization of SEN students

We will use the same measurement tool as for SEN students mentioned above to assess the other-dehumanization. Items are identical, except participants will rate the extent to which they perceive the humanness traits best describing their “SEN classmates/students/children”.

##### Prejudice

A 24-item measure will be used to assess participants’ prejudice towards SEN students in four dimensions (harm, separate, dependence, and idealization) [31]. It has previously been used to measure public prejudice towards people with developmental disabilities, and the average Cronbach’s ɑ > 0.75. Sample items include “I prefer not to interact with people who have SEN”. In addition, we will include a 5-item Social Distance Scale, Cronbach’s ɑs > 0.9 [47]. Sample items include, “I can accept SEN students to be my neighbours”. All items are rated on a 5-point Likert scale (1 = not at all, 5 = very much).

##### Subjective well-being

The same measurement as mentioned in the above section.

##### School functioning

The same measurement as mentioned in above section. Except for teachers, the questions assess their perception of the school functioning of SEN and non-SEN students in their class; and for parents, the questions assess their perception of the school functioning of their children.

##### Demographic information

It is similar to those mentioned in the above section.

In Study 1b, non-SEN students (enrolled in schools or classes without SEN peers), their parents, and university students will be invited to complete a Chinese version of the dehumanization measure with instruments described below.

##### Other-dehumanization of SEN students

Items are similar to those for the non-SEN peers, teachers and parents in Study 1a, but the target of dehumanization will be “SEN students” in general.

##### Prejudice

The same measurement as used in Study 1a.

##### School functioning

The same measurement as used in Study 1a.

##### Public policy support

It will include a 6-item measure to assess policy support for SEN students. The measurement will be adapted from a previous study regarding the dehumanization of low-SES groups and tap into several kinds of social welfare policy, Cronbach’s ɑs > 0.8 [38]. Sample items include “I support the government increasing healthcare spending for SEN students”. Items are rated on a 7-point Likert scale (1 = strongly disagree, 7 = strongly agree).

##### Demographic information

Similar to mentioned in Study 1a.

#### Phase 2

In Study 2a, we will invite SEN students, their parents, teachers, and non-SEN peers to a computer lab to participate in the experiment by completing a series of tasks on computers; and in Study 2b, we will similarly invite non-SEN university students and their parents to a computer lab for the same purpose. The procedures of both studies are described as follows.

- Participants will be informed that they are participating in a project to “help psychologists accurately categorize personality descriptors”, and once agreed, they will be randomly assigned into one of the four conditions that implement an HU (Human Uniqueness) manipulation or an HN (Human Nature) manipulation: lacking HU condition, having HU condition (as the control to lacking HU condition), lacking HN condition, or having HN condition (as the control to lacking HN condition). Thus, the current study is a four-condition between-subject experimental design.
- Participants in all conditions will first complete a humanness measure that rate “people in Hong Kong” in HU and HN on a 7-point Likert scale. These items are similar to the ones used in Phase 1, to serve the purpose of our cover story. In the meantime, they also establish the baseline of humanness attribution of each participant.
- Next, depending on the condition they were assigned, participants will read a paragraph of descriptions with tables or graphs addressing a fake study of how many, or the degree of, humanness traits SEN individuals demonstrate (Samples for the having HU and HN condition are attached in Tables 2 and 3; the materials for SEN students and their close ones will be tailor-made to sound more natural and realistic compared to the version for the public).
- After reading the paragraph, participants will complete the manipulation check questions. One question will ask to what extent they agree to the paragraph, and a few items assess the self-dehumanization (for SEN students) or other-dehumanization of SEN individuals (for other groups).
- Next, depending on their group identity, participants will complete a few questions similar to those used in Phase 1. Measurements include prejudice towards SEN students, social distance, public policy support, and subjective well-being.
- Lastly, participants will be debriefed regarding the purpose of the experiment.

**Table 2:** A sample reading material used in having HU condition

*Now, before answering more questions regarding a particular group in our society, please read the following description of the group adopted from a scientific report*
The member of SEN students in mainstream schools usually have few resources and are generally considered to have learning difficulties. However, the results of the study have shown that the member of this group tend to act according to their common sense, both good and bad, but very rational, and mostly have control over their behavior. Their civility and rational behaviors, as we understand it, are two of their main characteristics, according to the study. Additionally, their abilities to reflect on and control their actions give the impression that they are in fact mature, as they tend to behave logically

**Table 3:** A sample reading material used in having HN condition

*Now, before answering more questions regarding a particular group in our society, please read the following description of the group adopted from a scientific report*
The member of SEN students in mainstream schools usually have few resources and are generally considered to have learning difficulties. However, the results of the study have shown that the member of this group tend to act according to their emotions and feelings, both good and bad, very open, and sometimes have expressive behaviors. Their emotionality and cognitive openness, as we understand it, are two of their main characteristics, according to the study. Additionally, their abilities to reflect on and express their thoughts give the impression that they are in fact deep, as they tend to think in different ways

Prior to conducting Study 2c and 2d, we will produce three short video clips as priming materials. Two of the video clips will emphasize the humanness of SEN students (one for HU and one for HN). The video will incorporate the findings drawn from the Phase 1 and Study 2a and 2b into a script that highlights strength, humanness, and mental states, to make a vivid personality presentation that combines documentary, news reports, or interviews conducted by the research team (e.g., SEN students who have exceptional skills or kindness). The information in these two video clips will be comparable in amount, structure, and attractiveness. The third video clip will be used in the control condition, and contains only irrelevant and neutral information (e.g., a clip introduces cosmology).

Overall, the goal of producing these video clips is to adopt a popular format, deliver information via a layman's approach, and make priming accessible for future public education purposes.

In Study 2c, we will invite SEN students, their parents, teachers, and non-SEN peers; and in Study 2d, we will invite non-SEN individuals to a computer lab to complete a series of tasks.

- Participants will be randomly assigned into one of the three conditions: the HU emphasis condition, the HN emphasis condition, or the control condition. Thus, the current study is a three-condition between-subject experimental design.
- Participants will go through the same procedure as in Study 2a and 2b, with the exception that the reading material is replaced by a corresponding video clip mentioned in the above section.

### Data analysis

- ***Missing data handling***. To maximize the estimation efficiency and reduce bias, we will impute missing data using Markov Chain Monte Carlo multiple imputation methods [48] and k-Nearest Neighbor algorithm [49].
- ***Measurement model evaluation***. So far, there is no dehumanization measurement available specifically designed for SEN students. Thus, we will first evaluate the measurement model with coefficient Alpha, coefficient Omega, Confirmatory Factor Analysis, as well as Exploratory Structural Equation Modelling [50, 51].
- ***Associations of variables***. Structural Equation Modelling will be used to test the associations of dehumanization and outcome variables in Phase 1. We will apply the Actor-Partner Interdependence Model [52] to assess the dyadic effects between the self-dehumanization of SEN students and other-dehumanization from close relations (Fig. 1).
- ***Experimental effects and within-subject differences***. To address within-subject differences of dehumanization in Phase 1 and test the between-condition differences in Phase 2, we will conduct t-test and Analysis of Variance. Beyond evaluating the effect sizes (e.g., Cohen’s *d*), we will conduct Equivalence Test (53) and Bayesian statistical analysis to evaluate the robustness of the experimental effects.

## Discussion

The harmful consequences of undermining others’ humanity have been documented in history. The continued growing research into dehumanization, however, reveals its existence in our daily lives. Dehumanization is a complex and pervasive phenomenon across cultures, ethnic groups, and social hierarchies, and has a profound impact on moral judgment, prejudice, and public health. In recent decades, many researchers have dedicated great effort to understanding dehumanization and promoting awareness of it in a variety of contexts, such as sexual objectification, immigrants and refugees, and cultural differences. However, little attention has been paid to children or individuals with disabilities. In particular, the dehumanization of students with special education needs (SEN) in inclusive settings has been ignored. Furthermore, research studies attempting to view this from the victims’ perspective are lacking. Focusing on the self-dehumanization and other-dehumanization of SEN students, the study aims at investigating the prevalence and dynamics of dehumanization, identifying its negative consequences, and conducting experiments to reduce the dehumanization. The beneficiaries will include SEN students, non-SEN peers, teachers and parents. Findings will be with significant theoretical and empirical impacts.

### Theoretical impacts

The research will fill the research gap of a group previously neglected in dehumanization research. Previous dehumanization studies have mostly focused on ethnic and racial groups, as well as gender minorities and social minorities. However, little attention has been paid to inclusive settings. Few researches have been conducted among SEN students. Through surveying and conducting experiments among multiple stakeholders, the research will contribute to the literature by tapping into the prevalence and nature of the dehumanization of SEN students, trying to unfold the underlying mechanism of humanness attribution in human uniqueness and human nature, and furthermore, identifying the psychological consequences associated with it. In addition, validation of the tailor-made dehumanization measurement will enable the accessibility of the group and have a profound impact on future research. Teachers or researchers will find these validated tools useful for assessing the dehumanization and other-dehumanization toward students with special educational needs. The findings will help identify factors for further examination in Chinese community.

The study will offer a new perspective in dehumanization research, which examines the self- and other-dehumanization in a dyadic relationship model and widen our understanding of humanness among different minorities in history and society. Unlike most of the existing literature that has involved group-based investigations, the study will take the interpersonal approach to understand the dehumanization through the dyadic model. From such an integrative perspective, it will examine the impact of dehumanization within family units and within classrooms, seek to isolate the associations between SEN students and caregivers, non-SEN peers, and teachers. With the findings, it is possible to pinpoint such reciprocal relationships and shed lights to future research into how to intervene within the units to diminish dehumanization, especially among those close to SEN students, for realizing the inclusive community.

### Empirical impacts

Through experiments, the study will further the understanding of conditions under which emphasizing specific humanness buffers the negative consequences of other-dehumanization. The results will provide immediate feedback on the interventions, have important empirical impact on how educators, parents, and the public view SEN students, and incorporate such understanding into teaching, parenting, and improving public policy support for inclusive education. In addition, to increase the potential to generalize the experimental findings into practice, the study will include short video clips as priming materials, a method that is trending in popular culture (e.g., Instagram videos). The goal is to deliver information via a layman's approach and make priming accessible for future public education purposes. The resources will help seeking funding opportunities, e.g. Quality Education Fund, for program initiatives supporting SEN students and enhancing the community awareness in inclusion. At professional development level, the outcome can be further disseminated in teacher education courses or on-line teaching, particularly in SEN support and guidance, meeting the core value of the policy and practice of Whole School Approach to Integrated Education in Hong Kong mainstreaming schools.

The dyadic modelling will provide a vital view on how to ameliorate these negative consequences in family contexts and classroom contexts. By determining how SEN students are dehumanized by and identifying the process’s associations with close relations, the findings will offer insights not only to the “perpetrators” and the “victims”, but also to families, classrooms, and schools, facilitating finding solutions as a unit. In a broader picture, the study will enhance public awareness and provide insights to gain a deeper understanding of mental states and life obstacles SEN students encountered in their daily lives. By understanding the underlying mechanism and psychological impact of self-dehumanization and other-dehumanization, the outcome will contribute to future practical endeavours to ameliorate the dehumanization of SEN students, as well as other socioeconomically disadvantaged groups. The impact is significant in accumulating knowledge, skills and successful strategies in school, family and community support.

## Data Availability

Not applicable. This is an ongoing project, and the data collection is currently underway.
